# Improvement in obstructive sleep apnea after a tailored behavioural sleep medicine intervention targeting healthy eating and physical activity: a randomised controlled trial

**DOI:** 10.1007/s11325-017-1597-z

**Published:** 2017-12-08

**Authors:** Helena Igelström, Pernilla Åsenlöf, Margareta Emtner, Eva Lindberg

**Affiliations:** 1 Department of Neuroscience; Physiotherapy, Uppsala University, Box 593, BMC, SE-75124 Uppsala, Sweden; 2 Department of Medical Sciences; Respiratory, Allergy and Sleep Research, Uppsala University, Uppsala, Sweden

**Keywords:** Obstructive sleep apnea, Behaviour modification, Physical activity, Diet

## Abstract

**Purpose:**

The aim of the present single-centre randomised controlled trial was to assess the effect of a behavioural sleep medicine (BSM) intervention on obstructive sleep apnea (OSA) severity in patients who have been referred for new treatment with continuous positive airway pressure (CPAP).

**Methods:**

After baseline assessment including ventilatory and anthropometric parameters, and physical activity monitoring, 86 patients who were overweight (BMI ≥ 25) and had moderate-severe OSA with apnea-hypopnea index (AHI) ≥ 15 were randomised into a control group (CG; CPAP and advice about weight loss) or an experimental group (ExpG; CPAP and BSM intervention targeting physical activity and eating behaviour). The BSM intervention comprised 10 individual sessions with a dietician and a physiotherapist and included behaviour change techniques such as goal setting and self-monitoring. After 6 months, a new recording of ventilatory parameters was performed without CPAP.

**Results:**

In ExpG, 40% (*n* = 14) had improved from severe to moderate or mild OSA or from moderate to mild OSA compared to 16.7% in CG (*n* = 6, *p* = 0.02). Further, a lower AHI and amount body fat at baseline were correlated with improvement in severity class. Being in ExpG implied a mean improvement in AHI by 9.7 and an odds ratio of 4.5 for improving in severity classification.

**Conclusions:**

The results highlight the clinical importance of lifestyle modifications in conjunction with CPAP treatment in patients with OSA.

## Introduction

Obstructive sleep apnea (OSA) is a highly prevalent problem [1]. According to guidelines from the American Academy of Sleep Medicine [2], an apnea-hypopnea index (AHI) of 5–14.9 is defined as mild OSA, 15–29.9 is defined as moderate, while an AHI > 30 is defined as severe OSA. An AHI above 30 is correlated with higher risk of developing cardiovascular disease [3, 4] and thus, it is of clinical importance to find ways of reducing the respiratory events during sleep and the severity of the disease.

The first-line treatment for moderate-severe OSA is continuous positive airway pressure, CPAP [5]. The CPAP treatment has an instant effect on breathing during sleep and, in the longer run, decreases the risk for secondary problems such as cardiovascular disease and stroke [6, 7]. The majority of patients with moderate-severe OSA are overweight [8], and prevalence of obesity has been reported to be 34% [9]. Improvement of OSA has been reported of weight loss interventions [10–12], and weight loss is hypothesised to reduce the amount of visceral adiposity and abdominal pressure which in turn enables better diaphragmatic and ribcage movement leading to enhanced ventilation and fewer respiratory events [10]. Physical exercise has also been demonstrated to reduce AHI even with minimal changes in body weight [13, 14]. The causal links between exercise and respiratory events have not yet been fully understood, but one hypothesis refers to the reduction of rostral fluid shift during sleep [15].

Even though changes in diet and increased physical activity may not cure OSA, such behavioural changes may be clinically relevant to the individual by reducing the disease severity [10] and thereby the risk of comorbidities. Furthermore, the effect of such behavioural changes beyond the effects of CPAP treatment is unknown. The aim of the present study was to assess the effect of a behavioural sleep medicine (BSM) intervention targeting healthy eating and physical activity on OSA severity in overweight patients who were referred to begin treatment with CPAP due to moderate-to-severe OSA. Further, the aim was to identify baseline predictors for treatment success.

## Material and methods

### Design

**Fig. 1:**
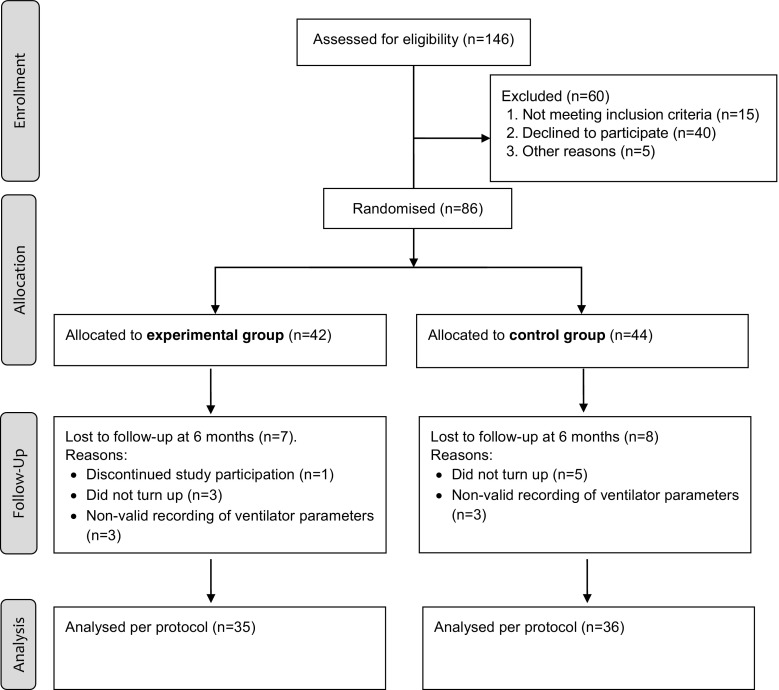
Flow chart of study participation from recruitment to follow-up at 6 months

The study was a prospective two-armed randomised controlled trial with follow-up at 6 months, evaluating the effects on OSA following a BSM intervention targeting physical activity and eating behaviour changes in patients with first-time CPAP treatment (Fig. 1). The data of the study were collected from March 2010 to March 2012. The study is registered at ClinicalTrials.gov (clinical trial number NCT01102920). Written consent was obtained from all the patients and the study was approved by the local ethics committee in Uppsala, Sweden (dnr 2009/004).

### Recruitment procedure

Patients considered eligible for study participation were all admitted to the sleep clinic at Uppsala University Hospital in Uppsala, Sweden. They were overweight (BMI ≥ 25) and diagnosed with OSA with an AHI of ≥ 15 and prescribed CPAP treatment. Participants were consecutively addressed by the examining physician, who gave written information about the study and asked about interest in participation in the study. Patients who volunteered were contacted by phone by a research assistant (HI) for an interview on leisure time physical activity. Those patients who were physically active less than the stated recommendations (150 min a week of moderate physical activity) were scheduled for an appointment to a study nurse for written consent and baseline measurement.

### Data collection methods

All measurements were made by a study nurse who was blinded to study group.

### Baseline assessments

#### Psychological and social factors

Sociodemographic data (age, gender, civil state, and education) was collected by questionnaires. Daytime sleepiness was assessed by the Epworth Sleepiness Scale [16], and depressive symptoms were assessed by the Montgomery Åsberg Depression Rating Scale [17].

#### Physical activity and sedentary time

To assess physical activity and sedentary time, participants wore an activity monitor (SenseWear™ Armband Pro 3 (BodyMedia Inc., Pittsburgh, PA, USA), SWA) during seven consecutive days at baseline and at a follow-up. The device contains a three-axis accelerometer that registers motion in addition to physiological responses to physical activity, such as galvanic skin response, skin temperature, and amount of heat dissipating from the body. The cut-off points defining moderate and vigorous intensity of physical activity were three and six metabolic equivalents (METs), respectively, whereby one MET equals energy expenditure at rest. METs of 1.5 or less was considered sedentary time. In addition, moderate-to-vigorous intense physical activity (MVPA; greater than or equal to 3METs), was calculated. After removal of night sleep, the number of steps and time spent in physical activity and sedentary activity, respectively, were calculated using SenseWear Professional v.8.1 (BodyMedia Inc., Pittsburgh, PA, USA) computer software. Data were considered valid when they contained ≥ 10 h of data from ≥ 90% of waking time for at least 4 days [18].

### Measurements at baseline and follow-up (6 months)

#### Ventilatory parameters

The primary outcome, AHI, was assessed by ambulatory recordings using the type 3 device Embletta (Embla systems) at baseline and follow-up. Signals recorded included nasal pressure through a nasal cannula attached to a pressure transducer, oronasal thermistor, pulse oximetry for oxyhemoglobin saturation, respiratory effort through piezoelectric belts, and body position. At the follow-up, the participants were instructed not to use the CPAP device for three consecutive nights before the night of recording. The recordings were scored and analysed blindly by a physician specialised in respiratory and sleep medicine (EL). Apneas were defined as cessation of airflow in nasal pressure for at least 10 s with continuing abdominal and thoracic movements while hypopneas were defined as ≥ 50% reduction in baseline airflow for at least 10 s in combination with an oxygen desaturation of ≥ 3% [2]. Data were considered valid when registration time reached or exceeded 240 min recording time. AHI was calculated as the number of apneas and hypopneas per hour of sleep.

#### Anthropometric data

Included anthropometric variables were weight (in kilogrammes), height (in metres), body mass index (BMI, kg/m^2^), waist circumference (in centimetres), neck circumference (in centimetres), sagittal abdominal diameter (in centimetres), and amount of body fat (denoted by percentage). All measurements were performed in light clothing without shoes.

Neck circumference was measured upright with a measuring tape round the neck just below larynx. Waist circumference was measured at standing, and the sagittal abdominal diameter was measured lying down with the back against the surface beneath [19]. Both waist circumference and sagittal abdominal diameter were measured midway between the lower rib margin and the anterior superior iliac crest at the end of normal exhalation.

Amount of body fat was measured by Bio-electric Impedance Analysis (BIA) using a Tanita BC-418MA Body Composition Analyser (Tanita Corporation of America, Arlington Heights, IL, USA). All numbers were rounded off to one decimal place.

### Measurement at follow-up (6 months)

#### CPAP adherence

At follow-up, CPAP usage was assessed by a questionnaire comprising questions about how many hours per night and how many nights every week participants had used their CPAP. Information about compliance to CPAP treatment was also extracted from medical records. CPAP usage was categorised into two groups: “non-compliant” defined as a mean CPAP usage < 4 h/night, and “compliant “was defined as CPAP usage ≥ 4 h/night.

### Randomisation procedure

After the baseline assessment, participants were randomly allocated either to the control group or the experimental group by a senior researcher not involved in the assessment procedures (PÅ). The randomisation procedure was made in blocks of 10 using a random integer generator (www.random.org).

### Interventions

#### Control group (CPAP regimen and advice)

All the patients were verbally informed about the association between being overweight and sleep apnea and about the aim of the CPAP treatment. They were all recommended to lose weight and increase physical activity, but no behavioural goal setting, action plans, or functional behavioural analyses were provided. The patient then met a CPAP nurse and was taught how to use the CPAP device. The correct CPAP pressure was titrated by an auto-CPAP during 5–14 nights at home followed by a visit to the CPAP nurse when the final CPAP pressure was decided.

#### Experimental group (CPAP regimen and a tailored behavioural sleep medicine intervention)

Regular CPAP treatment was provided in an identical manner as the controls. In addition, a BSM intervention targeting enhanced physical activity and sound eating habits was applied. The intervention was based on health psychology theories such as the constructs of self-determination [20] and self-regulation [21] and was applied in a stepwise manner. Based on each participants’ possibility to visit the hospital, eight to ten therapist-guided individual sessions were scheduled for the first 6 months and were gradually reduced in frequency. The treatment focused on regular food habits and on unsupervised home-based physical activities and the patients themselves managed the principal part of the treatment through homework assignments. The behavioural intervention comprised seven components, to be employed by each participant and in both physical activity behaviour and eating behaviour:

1. Preparation for action and motivational analysis
2. Goal setting (behaviour)
3. Action planning
4. Self-monitoring
5. Review of behavioural goals, action plans, and functional behavioural analysis
6. Barrier identification/problem solving
7. Maintenance and relapse prevention/coping planning

The same *types* of intervention components were used for all individuals in the experimental group, but the *content* was adjusted to individual prerequisites. Goals, action plans, and homework assignments were continuously formulated in a progressive manner with the aim of successively increased self-management by the patient. The details of the components of the behavioural sleep medicine intervention have been described elsewhere [22]. Weight was measured at three of the sessions.

The median number of session for all the participants in the experimental group was 9 (min, 1; max, 11). All the participants received the first component, *preparation for action and motivational analysis.* Six patients declined participation in the intervention; four of them withdrew within the first three sessions and thus did not complete the components of *goal setting (behaviour)*, *action planning*, and *self-monitoring.* The other two participants declined participation after 3 months, but before the completion of *maintenance and relapse prevention/ coping planning*. Eighteen participants (42.6%) completed all seven components including *maintenance and relapse prevention*, and 38 participants (90.5%) completed the first six components.

### Statistical analyses

All data are presented in numbers and proportions (%), or in means or medians with accompanying dispersion measurements (standard deviations (SD) or interquartile range (IQR), respectively). The significance level was set at α = 0.05.

OSA severity was categorised into mild (AHI 5– < 15), moderate (AHI 15– < 30), or severe (AHI greater or equal to 30) [23]. Comparative analyses were performed for those who had improved or not according to changes in AHI and OSA category at follow-up. For continuous and normally distributed variables, comparative analyses were made using the Student *t* test. For data with non-normal distribution and non-parametrical and categorical data, analyses were made using the Mann-Whitney *U* test and a chi-square test (or Fischer’s exact test), respectively.

Patients who improved in OSA category were compared to those who did not improve. Those variables in the in-between-group comparisons with a *p* value of < 0.1 were used in regression analyses with the following dependent variables:

- Change in AHI from baseline to follow-up (continuous variable)
- Improvement or not according to OSA category (categorical variable)

For the continuous dependent variable, a multiple linear regression was used, while a multiple logistic regression model was used for the categorical variable. To check for multicollinearity, the variance inflation factor (VIF) was calculated. The VIF for the independent variables was acceptable (range 1.019–1.021), suggesting there was no multicollinearity.

All analyses were done on an intention-to-treat basis, in that all participants with valid data on both assessments were included in the analysis even in cases of drop-outs. Missing data on AHI were not imputed, entailing a per protocol analysis.

## Results

Eighty-six participants entered the study averaging 54.9 ± 11.8 years of age, a BMI of 34.1 ± 5 kg/m^2^, and a mean AHI of 43.5 ± 20.7 (Table 1). The study groups did not differ at baseline, indicating a successful randomisation. At follow-up, data from 71 participants were analysed (Fig. 1).

**Table 1:** Participants’ characteristics at baseline. Numbers are presented in mean and standard deviation (SD), median and interquartile range (IQR), or frequency and percent (%)

	All (*n* = 86)	Experimental group (*n* = 42)	Control group (*n* = 44)
Age, mean (SD)	54.9 (11.8)	57.3 (12.1)	52.5 (11.2)
Gender, no (%)			
Male	71 (82.6)	36 (85.7)	35 (79.5)
Female	15 (17.4)	6 (14.3)	9 (20.5)
Civil state, no (%)			
Married/cohabitant	66 (76.7)	30 (71.4)	36 (81.8)
Living apart	3 (3.5)	1 (2.4)	2 (4.5)
Single/living alone	17 (19.8)	11 (26.2)	6 (13.6)
Education, no (%)			
Elementary school	20 (23.3)	10 (23.8)	10 (22.7)
High school	46 (53.5)	23 (54.8)	23 (52.3)
College	20 (23.3)	9 (21.4)	11 (25)
Occupation, no (%)			
Working	61 (70.9)	26 (61.9)	35 (79.5)
Retired	19 (22.1)	12 (28.6)	7 (15.9)
On sick leave	1 (1.2)	0	1 (2.3)
On sickness benefit	4 (4.7)	3 (7.1)	1 (2.3)
Smoking, no (%)			
Never smoked	37 (43)	16 (38.1)	21 (47.7)
Quit smoking	35 (40.7)	20 (47.6)	15 (34.1)
Smoking	14 (16.3)	6 (14.3)	8 (18.2)
Apnea-Hypopnea Index (AHI), mean (SD)	43.5 (20.7)	41.3 (19.8)	45.6 (21.5)
OSA category, no (%)			
Moderate (AHI 15–29.9)	30 (34.9)	15 (35.7)	15 (34.1)
Severe (AHI > 30)	56 (65.1)	27 (64.3)	29 (65.9)
Epworth Sleepiness Scale, median (IQR)	13 (6)	13 (7)	13 (7)
Body mass index (BMI), mean (SD)	34.5 (5.1)	34.8 (5.6)	34.1 (4.6)
Amount of body fat (%), mean (SD)	33.3 (6.3)	33.6 (6)	33 (6.7)

### Change in AHI from baseline to follow-up

From baseline to follow-up, participants in the experimental group reduced their AHI (−9.9, ±13) more than the control group (−1.9, ±19.9; *p* = 0.050).

### Change in OSA category from baseline to follow-up

**Fig. 2:**
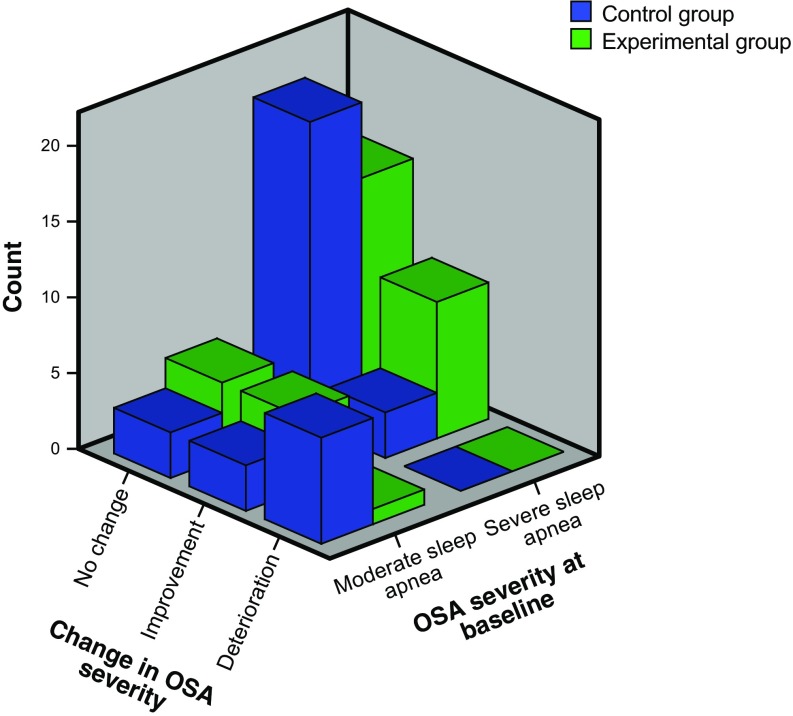
Number of participants in each study group having improved, deteriorated, or not changed at all in categorisation of obstructive sleep apnea (OSA) severity from baseline to follow-up at 6 months

An improvement in OSA category from severe to moderate or mild OSA or from moderate to mild OSA was more common in the experimental group (*n* = 14 vs. *n* = 6), *p* = 0.02 (Fig. 2). Those who had improved in OSA category were characterised by lower baseline values of AHI and amount of body fat, while there were no significant differences between the groups in CPAP compliance (Table 2). A deterioration to a more severe OSA category was found in 1 (2.9%) in the experimental group and in 7 (19.4%) of the controls (*p* = 0.019).

**Table 2:** Descriptive data for participants having improved in OSA category or not at follow-up. Numbers are presented in mean and standard deviation (SD) unless stated otherwise

	Improved (*n* = 20)	No change/deteriorated (*n* = 51)	*p*
Age (years)	56 (12.1)	54.2 (12.3)	0.596
Study group, no (%)			
Intervention	14 (70)	21 (41.2)	
Control	6 (30)	30 (58.8)	*0.029*
Gender, no (%)			
Male	17 (85)	42 (82.4)	
Female	3 (15)	9 (17.6)	0.789
Apnea-Hypopnea Index at baseline	32.9 (11.4)	48.1 (21.6)	*0.004*
Body Mass Index (kg/m^2^) at baseline	33.4 (5.6)	34.6 (5)	0.391
Neck circumference (cm) at baseline	43.1 (3.4)	43.4 (4.4)	0.810
Waist circumference (cm) at baseline	115.7 (14.7)	118.7 (13.3)	0.413
Percent body fat (%) at baseline	30.2 (5.9)	34.3 (5.8)	*0.009*
Daily average minutes of MVPA at baseline	*n =* 20	*n =* 43	
	76 (45)	82 (60)	0.715
Daytime sleepiness at baseline	12.5 (5.2)	12 (4.4)	0.763
Depressive symptoms at baseline	8.9 (5.7)	9 (7.1)	0.768
CPAP adherence at follow-up, no (%)	*n =* 20	*n =* 48	
At least 4 h per night	11 (55)	29 (60.4)	
Less than 4 h per night	9 (45)	19 (39.6)	0.789

### Multivariate analyses

#### Predictors of improvement in OSA category

The odds of improving in OSA category was 4.5 times higher in the experimental group after adjusting for baseline AHI and amount of body fat (*p* = 0.023). However, a higher AHI at baseline reduced the odds of improving in severity classification (*p* = 0.017), likewise in the case of a higher amount of body fat (*p* = 0.011; Table 3).

**Table 3:** Results from multiple logistic regression of study group, AHI (baseline) and amount body fat (baseline) on improvement in OSA category from baseline to follow-up at 6 months

Independent variable	B	SE	*p* value	OR	95% CI for OR	
					Lower	Upper
Study group	1.51	0.665	0.023	4.51	1.226	16.586
AHI at baseline	− 0.05	0.021	0.017	0.95	0.911	0.991
Percent body fat at baseline	− 0.16	0.065	0.011	0.85	0.747	0.963

#### Predictors of AHI improvement

According to multivariate regression analysis, study group, baseline AHI, and amount of body fat together explained 14.4% of the variance of AHI change from baseline to follow-up (*F* = 4.923 (df 3), *p* = 0.004). Amount body fat at baseline did not significantly contribute to the model (Table 4).

**Table 4:** Results from multiple linear regression of study group, AHI (baseline) and amount body fat (baseline) on change in Apopnea-Hypopnea Index (AHI) from baseline to follow-up at 6 months

Independent variables	*b*	SE	*p* value	95% CI for *b*	
				Lower	Upper
Study group	− 9.686	3.828	0.014	− 17.328	− 2.045
AHI at baseline	− 0.278	0.094	0.004	− 0.466	− 0.089
Percent body fat at baseline	0.490	0.316	0.125	− 0.139	1.120

Results are adjusted for all variables in the table

*b* = non-standardised regression coefficient; *SE* = standard error for *b*; *CI* = confidence interval for *b*

## Discussion

The results of the present study indicate that the BSM intervention in patients prescribed with CPAP therapy for moderate-severe OSA has a positive effect on OSA severity. The experimental group had a 4.5 times higher likelihood of having improved in OSA after 6 months and the improvement was not influenced by adherence to CPAP therapy.

The BSM intervention in the present study comprised both physical activity and healthy eating, whereas it is hard to say what contributed the most to the intervention effect. Still, the combination of physical activity and diet in patients with sleep apnea seems extra promising in reducing disease severity [10–12]. Thus, it is important to find ways of guiding patients in weight reduction, enhanced physical activity, and thereby reduce the respiratory events during sleep.

Identifying variables with potentially predictive power for change in OSA status might guide the clinician and the patient to make adequate decisions. In the present study, a *lower* amount of body fat at baseline was correlated with a higher chance of being classified with a milder OSA severity at follow-up than at baseline. It may be that in persons who are only modestly overweight (i.e. those who do not have such a high amount of body fat), only small anthropometrical changes are needed in order for effects to be noticeable on sleep registrations and in turn, OSA severity categorisation.

Having a higher AHI at baseline was associated with larger reductions in AHI, which has also been reported in a meta-analysis by Araghi et al. [10], where a linear relationship was seen between baseline AHI and reduction in AHI. However, those with higher baseline AHI and amount of body fat were less likely to improve enough in AHI to cause an improvement in OSA category. Thus, it seems that the BSM intervention was of most benefit to those with lower AHI and lower amount of body fat at study entry, at least when it comes to improvements in OSA category. For those with a high AHI at baseline, or with severe obesity, the pronounced changes required to improve OSA category might not be possible to obtain during a 6-month period. It might also be that a more intense intervention and longer follow-up period is needed for effects to be seen in patients with a higher AHI and amount of body fat.

The BSM intervention in the present study was based on individual prerequisites and goals. Thus, the diet for an individual could comprise caloric restriction, exchanging saturated fat into unsaturated fat, or reducing the intake of soft drinks, etc. Other dietary trials in patients with moderate-severe OSA have investigated the effect of weight reduction on AHI by means of a very low-calorie diet (VLCD) and meal replacements, accompanied by faster and greater weight reduction and subsequent improvements in AHI [24]. Another alternative is bariatric surgery [25], which, like VLCD and meal replacement, often implies a faster weight reduction. In summary, there are many ways of reducing weight in patients who are overweight and suffer from OSA, which implies a more multifaceted clinical praxis. Future studies should study whether a more intensified behavioural intervention (e.g. supervised physical exercise and more progressive goal setting in eating habits) may lead to behavioural changes large enough for effects to be observed in both AHI and OSA severity.

The importance of reducing AHI in severe OSA is important, due to the elevated risk of developing cardiovascular disease [26]. Reductions in AHI may be achieved by different means such as CPAP, mandibular devices, dietary changes, and enhanced physical activity, and a patient-centred care considering different treatment options is important for engagement and treatment adherence [27]. Due to the challenges of initiating CPAP therapy [28], changing eating habits [29] and physical activity [30], it is important in clinical praxis to tailor treatment to individual prerequisites.

### Methodological considerations

Even though patients with greater severity at baseline experienced a larger reduction in AHI in the present study, the improvement in these patients was not large enough to help the patients improve in OSA category. It might be that the OSA categorisation is too coarse and insensitive to change, especially in the “*Severe*” category. However, since AHI and OSA categorisation are often used in combination in clinical praxis, both outcomes were chosen to enable a multifaceted analysis. It is worth highlighting that even if an individual at follow-up is still classified as having severe OSA, the improvements seen in continuous values of AHI from baseline to follow-up may still be of important clinical significance for the individual [11].

## Conclusion

The results indicate that the BSM intervention is effective for improving patients’ AHI and OSA category. However, the present data do not conclude the mechanisms behind the intervention effect. A higher AHI at baseline was correlated with larger reductions in AHI over time, even though these changes where not large enough to cause a transition from one OSA category to another. Rather, the higher AHI and amount of body fat at baseline, the more likely there is a need for more intensified or longer interventions in order for such effects to occur. The results highlight the clinical importance of lifestyle modifications in addition to CPAP treatment in patients with OSA, but the question about how to increase the effectiveness of such interventions still remains.
